# Diabetic Retinopathy: Pathophysiology and Treatments

**DOI:** 10.3390/ijms19061816

**Published:** 2018-06-20

**Authors:** Wei Wang, Amy C. Y. Lo

**Affiliations:** Department of Ophthalmology, Li Ka Shing Faculty of Medicine, The University of Hong Kong, Hong Kong, China; u3003921@hku.hk

**Keywords:** vascular pathology, inflammation, retinal degeneration, anti-VEGF, laser treatment

## Abstract

Diabetic retinopathy (DR) is the most common complication of diabetes mellitus (DM). It has long been recognized as a microvascular disease. The diagnosis of DR relies on the detection of microvascular lesions. The treatment of DR remains challenging. The advent of anti-vascular endothelial growth factor (VEGF) therapy demonstrated remarkable clinical benefits in DR patients; however, the majority of patients failed to achieve clinically-significant visual improvement. Therefore, there is an urgent need for the development of new treatments. Laboratory and clinical evidence showed that in addition to microvascular changes, inflammation and retinal neurodegeneration may contribute to diabetic retinal damage in the early stages of DR. Further investigation of the underlying molecular mechanisms may provide targets for the development of new early interventions. Here, we present a review of the current understanding and new insights into pathophysiology in DR, as well as clinical treatments for DR patients. Recent laboratory findings and related clinical trials are also reviewed.

## 1. Introduction

Diabetic retinopathy (DR) is a major complication of diabetes mellitus (DM), which remains a leading cause of visual loss in working-age populations. The diagnosis of DR is made by clinical manifestations of vascular abnormalities in the retina. Clinically, DR is divided into two stages: non-proliferative diabetic retinopathy (NPDR) and proliferative diabetic retinopathy (PDR). NPDR represents the early stage of DR, wherein increased vascular permeability and capillary occlusion are two main observations in the retinal vasculature. During this stage, retinal pathologies including microaneurysms, hemorrhages and hard exudates can be detected by fundus photography although the patients may be asymptomatic. PDR, a more advanced stage of DR, is characterized by neovascularization. During this stage, the patients may experience severe vision impairment when the new abnormal vessels bleed into the vitreous (vitreous hemorrhage) or when tractional retinal detachment is present. The most common cause of vision loss in patients with DR is diabetic macular edema (DME). DME is characterized by swelling or thickening of the macula due to sub- and intra-retinal accumulation of fluid in the macula triggered by the breakdown of the blood-retinal barrier (BRB) [1]. DME can occur at any stage of DR and cause distortion of visual images and a decrease in visual acuity. Current treatment strategies for DR aim at managing the microvascular complications, including intravitreal pharmacologic agents, laser photocoagulation and vitreous surgery. Intravitreal administration of anti-VEGF agents is currently the mainstay of therapy for both early and advanced stages of DR. While the conventional laser therapy only provides stabilization of visual acuity, anti-VEGF therapy can result in visual improvement with less ocular adverse effects. However, according to the Diabetic Retinopathy Clinical Research Network (DRCR.net) study (Protocol I), ≥3-line improvement in best-corrected visual acuity (BCVA) was achieved in only 29% of DME patients receiving two years of anti-VEGF treatment [2]. The inadequate response to anti-VEGF may be associated with the involvement of other molecular pathways than VEGF during the pathogenesis of DR. Studies investigating the underlying mechanisms of DR are of great importance, which may provide potential targets for the development of new alternative treatments. Here, we present a brief overview of current understanding of and new insights into the pathophysiology of DR. Novel therapeutic targets and potential pharmacological agents being tested in clinical trials are also discussed.

## 2. Pathology in DR

### 2.1. Hyperglycemia and Retinal Microvasculopathy

DR has long been recognized as a microvascular disease. Hyperglycemia is considered to play an important role in the pathogenesis of retinal microvascular damage. Multiple metabolic pathways have been implicated in hyperglycemia-induced vascular damage including the polyol pathway, advanced glycation end products (AGEs) accumulation, the protein kinase C (PKC) pathway and the hexosamine pathway [3].

The earliest responses of the retinal blood vessels to hyperglycemia are dilatation of blood vessels and blood flow changes. These changes are considered to be a metabolic autoregulation to increase retinal metabolism in diabetic subjects [4]. Pericyte loss is another hallmark of the early events of DR. Evidence of apoptosis of pericytes triggered by high glucose has been shown in both in vitro and in vivo studies [5,6]. Since pericytes are responsible for providing structural support for capillaries, loss of them leads to localized outpouching of capillary walls. This process is associated with microaneurysm formation, which is the earliest clinical sign of DR [7]. In addition to pericyte loss, apoptosis of endothelial cells and thickening of the basement membrane are also detected during the pathogenesis of DR, which collectively contribute to the impairment of the BRB [8]. Furthermore, pronounced loss of pericytes and endothelial cells results in capillary occlusion and ischemia. Retinal ischemia/hypoxia leads to upregulation of VEGF through activation of hypoxia-inducible factor 1 (HIF-1) [9]. Other evidence suggested that phospholipase A2’s (PLA2) elevation under the diabetic condition also triggers upregulation of VEGF [10]. VEGF, a key factor involved in the progression of PDR and DME, is believed to increase vascular permeability by inducing phosphorylation of tight junction proteins such as occludin and zonula occludens-1 (ZO-1) [11]. Moreover, as an angiogenic factor, VEGF promotes proliferation of endothelial cells through activation of mitogen-activated protein (MAP) [12]. Enhanced expression of VEGF has been detected in the retina of diabetic mouse, as well as the vitreous of patients with DME and PDR [13,14,15].

Other angiogenic factors such as angiopoietins (Ang-1, Ang-2) are also involved in the regulation of vascular permeability by interacting with endothelial receptor tyrosine kinase Tie2 [16]. Ang-2, antagonist of Tie2, has been shown to promote vascular leakage in the diabetic rat retina [17]. It is speculated that angiogenic factors besides VEGF might be involved in the alteration of microvasculature during DR; thus, they may provide novel therapeutic targets.

### 2.2. Inflammation

Inflammation plays an essential role in the pathogenesis of DR. Chronic low-grade inflammation has been detected widely in different stages of DR in both diabetic animal models and patients [18,19]. Leukostasis has been recognized as a key process in the early stage of DR. In 1991, Schröder et al. first reported the occlusion of retinal microvasculature by monocytes and granulocytes in streptozotocin (STZ)-induced diabetic rats [20]. Increased adherence of leukocytes was detected in the retinal vasculature as early as three days after induction of diabetes in rats [21]. The researchers also found that increased leukostasis is spatially correlated with endothelium damage and BRB impairment in diabetic rats [20]. Further studies demonstrated that leukostasis contributed to endothelial cell loss and breakdown of BRB through the Fas (CD95)/Fas-ligand pathway [22].

Leukocyte-endothelium adhesion mediated by adhesion molecules has been implicated in leukostasis in diabetes. Increased leukocyte adhesion and upregulated expression of leukocyte b2-integrins CD11a, CD11b, and CD18 were reported in diabetic rats and patients [23,24]. Additionally, endothelial cell adhesion molecules such as intercellular adhesion molecule-1 (ICAM-1), vascular cell adhesion molecule (VCAM)-1 and selectins (E-selectin) are also found to be increased in diabetic animals and patients [18,25,26] Expression of VCAM-1 and E-selectin in the plasma of patients is correlated with the severity of DR [18]. Genetic deficiency of CD18 or ICAM-1 resulted in significantly reduced adherent leukocytes [27]. Inhibition of CD18 or ICAM-1 with anti-CD18 F(ab9)2 fragments or antibody decreased retinal leukostasis and vascular lesions in diabetic rats [18,27].

Chemokines, which regulate the attraction and activation of leukocytes, have also been shown to be involved in the pathogenesis of DR. Chemokines such as monocyte chemotactic protein-1 (MCP-1), macrophage inflammatory protein-1alpha (MIP-1α), and MIP-1β have been reported to be elevated in diabetic patients [28]. MCP-1 deficiency leads to reduced retinal vascular leakage in diabetic mice [29]. Furthermore, inflammatory cytokines such as tumor necrosis factor alpha (TNF-α), interleukin 6 (IL-6), IL-8 and IL-1β were significantly upregulated in diabetic patients, and their expression level was correlated with the severity of DR [30,31].

Retinal glial cell dysfunction is also presumed to be involved in the initiation and amplification of retinal inflammation in DR [32]. Glial cells in the retina including astrocytes, Müller cells and microglia are responsible for providing structural support and maintaining homeostasis in the retina [33]. Under hyperglycemic stress, microglia is activated, followed by increased secretion of TNF-α, IL-6, MCP-1 and VEGF [32]. Later involvement of Müller cells and astrocytes is associated with the amplification of inflammation responses by producing proinflammatory cytokines [33].

### 2.3. Retinal Neurodegeneration

Retinal neurodegeneration is an early event during the progression of DR. Apoptosis of retinal neurons can be observed in diabetic rats as early as one month after induction of diabetes [34]. Upregulation of pro-apoptotic molecules such as cleaved caspase-3, Bax and Fas has been detected in retinal neurons in diabetic animals and subjects [35,36,37]. Mitochondrial dysfunction has been implicated in retinal degeneration in DR. In donor eyes of diabetic subjects, retinal expression of pro-apoptotic mitochondrial proteins such as cytochrome c and apoptosis-inducing factor (AIF) were found to be significantly increased [37]. In vitro studies demonstrated that high glucose exposure was associated with increased mitochondrial fragmentation and cell apoptosis [38]. In addition to mitochondrial damage, involvement of oxidative stress in diabetes-induced retinal degeneration has also been widely investigated. In the diabetic mouse retina, reactive oxygen species (ROS) generation is significantly increased [39]. Suppression of ROS generation effectively inhibited visual impairment and caspase-3-mediated retinal neuronal apoptosis [39].

There is growing evidence that retinal neurodegeneration may be an independent pathophysiology of DR. In a mouse model of diabetes, loss of ganglion cells and reduction in retinal thickness were observed preceding the presence of microvascular alterations [40]. In diabetic patients, inner retinal thinning was detected with no DR or minimal DR (microaneurysms) [40,41]. Therefore, further investigation of the molecular mechanisms underlying retinal neurodegeneration may provide potential therapeutic targets for early intervention in DR.

## 3. Current and Novel Treatment Strategies in DR

### 3.1. Anti-Angiogenic Therapy

#### 3.1.1. Anti-VEGF Drugs

The advent of anti-VEGF therapy has revolutionized the treatment of DR. Currently, anti-VEGF drugs that have been tested in clinical trials for DR treatment include the U.S. Food and Drug Administration (FDA)-approved pegaptanib (Macugen, OSI/Eyetech, New York, NY, USA), ranibizumab (Lucentis, Genentech, Inc., South San Francisco, CA, USA), aflibercept (EYLEA; Regeneron, Tarrytown, NY, USA) and the off-label intravitreal bevacizumab (Avastin, Genentech, Inc., South San Francisco, CA, USA) (Table 1). Among these agents, ranibizumab is the most comprehensively evaluated in clinical trials such as the Diabetic Retinopathy Clinical Research Network (DRCR.net), RISE (clinicaltrials.gov ID: NCT00473330), RIDE (clinicaltrials.gov ID: NCT00473382), RESOLVE (clinicaltrials.gov ID: NCT00284050), RESTORE (clinicaltrials.gov ID: NCT00687804), etc. The results of the RESOLVE and RESTORE studies showed that greater gains in BCVA were obtained with intravitreal ranibizumab when compared with laser monotherapy in patients with clinically-significant DME [42,43]. In the VISTA (clinicaltrials.gov ID: NCT01363440) and VIVID (clinicaltrials.gov ID: NCT01331681) trials, intravitreal aflibercept was associated with better visual outcomes than standard laser therapy in patients with DME [44]. The DRCR.net Protocol S (clinicaltrials.gov ID: NCT01489189) and CLARITY (trial registration number: ISRCTN 32207582) studies showed that anti-VEGF agents were also beneficial in the treatment of PDR [45,46]. Moreover, the results of the DRCR.net Protocol T (clinicaltrials.gov ID: NCT01627249) trial showed that aflibercept was superior to ranibizumab and bevacizumab in improving visual acuity for the treatment of patients with moderate or worse initial visual acuity loss [47].

However, limitations and adverse effects of anti-VEGF therapy are also of great concern. Due to the short half-life time of anti-VEGF agents, monthly or bimonthly injections are needed to ensure efficacy. The incidence of endophthalmitis, a rare adverse effect of intravitreal injection, may be increased by frequent injections. In the DRCR.net Protocol I trial (five years), three cases (0.08%) of injection-related endophthalmitis were reported following 3973 injections [48]. Financial burden and the patients’ poor compliance also limited the use of anti-VEGF drugs in clinical practice. Moreover, while VEGF may play a neuroprotective role in the retina, the use of high-dose anti-VEGF drugs requires careful consideration [49].

#### 3.1.2. Other Anti-Angiogenic Drugs

**Figure 1:**
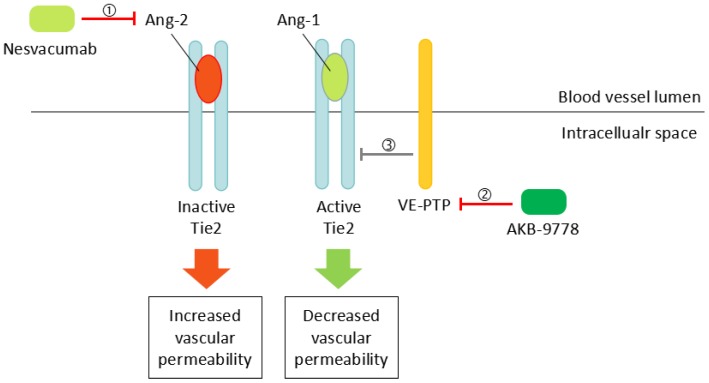
① Nesvacumab activates Tie-2 signaling and decreases vascular permeability by inhibiting Ang-2, an antagonist of Tie2. ② AKB-9778 activates Tie-2 signaling by inhibiting ③ VE-PTP, a negative regulator of Tie-2.

Currently, several anti-angiogenic drugs besides anti-VEGF agents are under clinical investigations (Table 1). Squalamine demonstrated better visual recovery than control groups in patients with macular edema by inhibiting multiple angiogenic factors (VEGF, PDGF, b-FGF) [50]. Clinical study testing the effect of squalamine in combination with ranibizumab in patients with DME is in progress (clinicaltrials.gov ID: NCT02349516). Novel agents targeting players in the Ang-Tie2 signaling pathway (Section 2.1) have also been developed. AKB-9778 is a small molecule that blocks the negative Tie2 regulator vascular endothelial-protein tyrosine phosphatase (VE-PTP); therefore, it activates Tie2 and decreases vascular permeability (Figure 1) [51]. A clinical trial investigating AKB-9778 in the treatment of DME is ongoing (NCT01702441). Nesvacumab is an inhibitor of Ang-2, which decreases vascular permeability by activating Tie2 (Figure 1). Nesvacumab co-formulated with VEGF inhibitor aflibercept is being tested in a phase 2 trial in DME patients (RUBY study, clinicaltrials.gov ID: NCT02712008). Another bispecific antibody RO6867461 targeting both Ang-2 and VEGF is also under test in DME patients (BOULEVARD study, clinicaltrials.gov ID: NCT02699450).

### 3.2. Anti-Inflammatory Therapy

#### 3.2.1. Intravitreal Steroid

Intravitreal corticosteroids have become increasingly important in the treatment of DME, especially in refractory DME and cases lacking a response to anti-VEGF therapy [53]. Refractory cases of DME and nonresponders to anti-VEGF are presumed to be driven by multiple cytokines. As potent anti-inflammatory agents, corticosteroids target a broad array of mediators involved in the pathogenesis of DME including VEGF, TNF-α, chemokines, leukostasis and phosphorylation of tight-junction proteins. Currently, intravitreal corticosteroids used in the clinical trials for DME treatment include the off-label triamcinolone acetonide, the FDA-approved dexamethasone (DEX) intravitreal implant and the fluocinolone acetonide (FA) intravitreal implant (Table 2).

In the DRCR Protocol I trial, intravitreal triamcinolone showed promising efficacy in improving visual acuity and reducing the thickness of central retina in patients with DME through the 24 week’s visit [48]. The effectiveness of triamcinolone started to decline after 24 weeks due to high risks of adverse ocular events including elevated intraocular pressure (IOP) and cataracts. During the two-year follow-up, elevated IOP was detected in 50% of patients, and cataract surgery was performed in 59% of patients in the triamcinolone group [48]. It is noted that for the treatment of DME in pseudophakic eyes, intravitreal triamcinolone demonstrated comparable effectiveness to that of ranibizumab.

The DEX intravitreal implant is a biodegradable delivery system that releases corticosteroids into the vitreous in a sustained manner for up to six months. The utility of the dexamethasone delivery system ensures prolonged drug exposure and remarkably reduces the frequency of injections, thus leading to better patient compliance. In the three-year study of the DEX implant (0.7 and 0.35 mg) in patients with DME, the mean number of injections over three years was four. Significantly greater BCVA improvement and reduction in central retinal thickness (CRT) were achieved in DEX implant groups when compared with the sham group [54]. Cataract-related adverse events were developed in more than 60% of patients in the DEX implant group [54]. In an 18-month study, the DEX implant (0.7 mg) significantly improved BCVA and reduced CRT in patients with refractory DME [55]. It is noted that anterior chamber dislocation is a rare complication of intravitreal implant, but the implant can be repositioned by injection of balanced saline solution [57].

Two FA implants have been tested in clinical trials for treating DME. Retisert (Bausch & Lomb, Inc., Rochester, NY, USA) is an FDA-approved 0.59-mg FA implant approved for treating chronic noninfectious posterior uveitis. In patients with persisting or recurring DME, Retisert is superior at improving BCVA when compared with standard therapy. However, the percentage of IOP elevation and cataract development is 61.4% and 91%, respectively [58]. Given the high percentages of ocular adverse effects of the Retisert implant, a 0.2-mg FA insert Iluvien (Alimera Sciences, Alpharetta, GA, USA) has been designed for the treatment of DME. Two models of Iluvien, which releases FA at a low dose of 0.23 and 0.45 μg/day, respectively, have been tested. In a two-year study, Iluvien (0.23 and 0.45 μg/day) successfully increased BCVA in patients with persistent DME with a single injection [56]. Currently, the 0.23-µg/day insert has been approved by the FDA for the treatment of DME, which is associated with a significantly lower rate of IOP elevation [56].

As described above, intravitreal corticosteroids showed promising efficacy in the treatment of DME. Particularly, sustained release of corticosteroids is associated with a lower frequency of intraocular injections, lower cost and better patient compliance. However, given the high incidence of adverse effects, corticosteroids are generally considered as a second-line option for patients insufficiently responding to other therapeutic treatments. Additionally, since the efficacy of corticosteroids in the treatment of PDR has not been determined, anti-VEGF agents should be used as a first-line therapy if the patient shows a high risk of progression to PDR.

#### 3.2.2. Non-Steroid Anti-Inflammatory Drugs

As one of the most important proinflammatory cytokines present in the vitreous of DR patients, IL-6 has been investigated as a promising target for anti-inflammatory therapy for DR. Antibodies against IL-6 (EBI-031) and the IL-6 receptor (tocilizumab) have been developed. Clinical trials have been carried out to test the efficacy and safety of EBI-031 (clinicaltrials.gov ID: NCT02842541) and tocilizumab (clinicaltrials.gov ID: NCT02511067) in patients with DME (Table 2). 

An antagonist of the adhesion molecule integrin is also under clinical investigation. Luminate (ALG-1001) is an integrin inhibitor that blocks multiple integrin receptors. It has demonstrated promising effects in alleviating swelling of the macula and promoting visual gain in a phase 2b trial for DME (DEL MAR study, clinicaltrials.gov ID: NCT02348918). A phase 2b trial PACIFIC (Available online: http://www.allegroeye.com/?s=PACIFIC) has been carried out to test the effect of (Table 2).

### 3.3. Laser Treatment

#### 3.3.1. Traditional Laser Treatments

Laser photocoagulation has been the gold standard for the treatment of both DME and PDR before the advent of anti-VEGF therapy. Focal/grid macular laser therapy was shown to effectively alleviate edema of the macula and reduced the risk of moderate visual loss by 50% in the three-year Early Treatment Diabetes Retinopathy Study (ETDRS) [59]. Panretinal photocoagulation (PRP) has also been used for the treatment of PDR and significantly reduced the risk of severe visual loss, especially in cases with high-risk complications such as vitreous hemorrhage [60]. The exact mechanisms by which laser therapy reduces DME and induces regression of neovascularization remains unclear. It is hypothesized that direct closure of leaking microaneurysms, the decrease of retinal blood flow associated with reduced retinal tissues and improved oxygenation, as well as stimulation of retinal pigment epithelium (RPE) might be implicated [61,62]. Given its destructive nature, laser therapy may cause permanent damage to the retinal cells, leading to side effects such as mild central visual loss and reduced night vision [63]. Although anti-VEGF therapy has increasingly become the mainstay of therapy for DR, laser therapy still plays an important role as an adjuvant treatment or rescue therapy. The utility of focal laser as an adjuvant treatment dramatically reduced the frequency of anti-VEGF injections when compared with anti-VEGF treatment alone in DME patients [64] (Table 3).

#### 3.3.2. New Laser Approaches

Nowadays, efforts have been made for the development of new laser approaches to reduce side effects. The pattern scanning laser (PASCAL) is a new laser method used for the treatment of DME and PDR. It reduces laser-induced retinal damage by providing a more precise control of the laser and a decrease in treatment time [65]. Micropulse techniques such as the subthreshold micropulse diode laser (D-MPL) have been employed to facilitate the delivery of subthreshold burns in order to minimize collateral damage [66]. More recently, the utilization of a navigated laser system (NAVILAS) further extended the accuracy of laser spots applied to the retina and resulted in favorable visual outcomes [67] (Table 3). In the future, developments in laser technology may further increase the safety and effectiveness of laser photocoagulation in the treatment of DR.

### 3.4. Other Therapeutic Agents

#### 3.4.1. Cardiolipin-Targeting Peptide (MTP-131)

Cardiolipin is a phospholipid in the inner mitochondrial membrane that might be involved in cell apoptosis [68]. MTP-131, a selective cardiolipin-targeting peptide, showed a protective effect on visual function in a diabetic mouse model by attenuating mitochondrial oxidative stress [69]. This indicated that cardiolipin may be important in mediating apoptosis of retinal neurons. A clinical trial (SPIOC-101, clinicaltrials.gov ID: NCT02314299) has been carried out to determine the effect of MTP-131 (Ocuvia™) topical ophthalmic solution in patients with DME (Table 4).

#### 3.4.2. Alpha-Lipoic Acid

Alpha-lipoic acid (ALA) is a mitochondria-specific antioxidant used in Alzheimer’s disease as a neuroprotective agent. It successfully prevented retinal ganglion cell loss and NFL thinning in an STZ-induced diabetic model [72]. ALA supplementation has been shown to be associated with improved visual acuity in patients with type 1 and type 2 diabetes [70] (Table 4).

#### 3.4.3. Lutein

Lutein, a member of the carotenoid family, is a potent antioxidant accumulated in the human retina. With its anti-oxidant, anti-inflammatory and neuroprotective properties, lutein has shown a promising effect in various retinal disease models [73,74]. In the diabetic mouse, lutein treatment effectively prevents retinal changes [75]. Moreover, lutein supplementation is associated with improved visual function in patients with NPDR [71] (Table 4).

#### 3.4.4. ARA290

ARA290 is a small erythropoietin (EPO)-derived peptide. EPO, a mediator of erythropoiesis, has been extensively shown to have a neuroprotective role in many animal models of neurodegeneration. In diabetic rats, ARA290 showed promising efficacy in treating DR by preventing neuroglial and vascular degeneration [76]. Currently, the effectiveness of ARA290 in the treatment of DME is under evaluation in a phase 2 clinical trial.

#### 3.4.5. Darapladib

Lipoprotein-associated phospholipase A2 (Lp-PLA2) has been shown to be involved in the damage of BRB during DR. Inhibition of Lp-PLA2 in diabetic rats significantly suppressed BRB breakdown. Therefore, Lp-PLA2 may serve as a therapeutic target for the treatment of DME [77]. Darapladib, a specific Lp-PLA2 inhibitor, demonstrated significant improvements in BCVA and macular edema in a three-month phase 2a study for the treatment of DME [78].

## 4. Conclusions

DR has long been known as a microvascular disease. Increasing laboratory and clinical evidence suggested that inflammation and retinal neurodegeneration may be implicated in DR as independent pathogenesis pathways. The development of agents targeting molecules in these pathways may provide new therapeutic treatments for DR. In the last decade, intravitreal anti-VEGF agents have become the first-line therapy for DME and PDR. However, in clinical practice, the use of anti-VEGF drugs is limited due to the requirement of frequent injections, financial burden and poor compliance of patients. In fact, laser photocoagulation still plays an important role in the treatment of DR as an adjuvant treatment. Intravitreal corticosteroids have demonstrated clinical benefits in the treatment of refractory DME or cases lacking response to anti-VEGF therapy. Yet, it remains difficult for patients with severe visual loss to achieve reading or driving vision with currently available therapeutics. Optimization of current treatment therapies regarding the number of intravitreal injections, dosage and duration, as well as of strategies for combination therapy is of great importance to improve the life quality of patients with DR. Further investigation is required to provide a better understanding of the pathogenesis of DR. Greater effort is needed to facilitate translation of recent research findings from the bench to the bedside.

## Figures and Tables

**Table 1:** Anti-angiogenic agents for the treatment of DR.

Classification	Drugs	Status for DR Treatment	Clinical Benefits	Adverse Effects
Anti-VEGF	Ranibizumab (Lucentis) [42,43]	FDA approved	Greater BCVA improvement and greater reduction in CRT over laser in treating DME (DRCR.net Protocol T, RESOLVE and RESTORE trials); non-inferior to PRP in treating PDR at two years (DRCR.net Protocol S)	a. Elevation in intraocular pressure b. Vitreous hemorrhage c. Inflammation [47]
	Pegaptanib (Macugen) [52]	FDA approved	Greater BCVA improvement over sham groups in treating DME (phase 2/3, multicenter, two-year trial)	a. Conjunctival hemorrhage b. Elevation in intraocular pressure
	Aflibercept (EYLEA) [44]	FDA approved	Greater BCVA improvement over laser in treating DME (VISTA, VIVID, DRCR.net Protocol T trials) and PDR (CLARITY trial)	a. Elevation in intraocular pressure b. Vitreous hemorrhage c. Inflammation [47]
	Bevacizumab (Avastin) [47]	Off-label use	Greater reduction in CRT and better median visual acuity over laser (DRCR.net Protocol T trial)	a. Elevation in intraocular pressure b. Vitreous hemorrhage c. Inflammation
Non-specific anti-angiogenic	Squalamine (inhibits VEGF and other growth factors) [50]	Phase 2 trial (clinicaltrials.gov ID: NCT02349516) in progress	-	-
	AKB-9778 (Tie2 activator) [51]	Phase 2 trial (clinicaltrials.gov ID: NCT01702441) in progress	Greater reduction in CRT in the combination group over ranibizumab monotherapy group (phase 2a clinical trial)	a. Diabetic retinal edema worse b. Visual acuity reduced
	Nesvacumab (Anti-Ang-2) (Section 3.1.2)	Clinical trial (RUBY trial) in progress	Results of phase 2 RUBY trial did not provide sufficient differentiation between the combined (nesvacumab + aflibercept) and aflibercept monotherapy treatments to warrant phase 3 development	No new safety signals observed when compared with other anti-angiogenic agents
	RO6867461 (bispecific antibody: anti-ang-2 + anti-VEGF)	Clinical trial (BOULEVARD trial) in progress (Section 3.1.2)	Greater adjusted BCVA improvement and greater reduction in CRT over ranibizumab in DME patients (phase 2 BOULEVARD trial)	Well tolerated with no new safety signals observed

**Table 2:** Anti-inflammatory drugs for the treatment of DR.

Classification	Drugs	Status for DR Treatment	Clinical Benefits	Adverse Effects
Intravitreal steroids	Triamcinolone [48]	Off-label use	Greater improvements in triamcinolone + prompt laser group over laser alone in pseudophakic eyes	a. Cataract surgery b. Elevation in intraocular pressure c. Vitreous hemorrhage
	DEX implant (Ozurdex) [54,55]	FDA approved	Greater BCVA improvement and greater reduction in CRT over sham group in patients with DME (three-year trial)	a. Cataract b. Elevation in intraocular pressure c. Vitreous hemorrhage
	FA insert (Iluvien, 0.2 mg) [56]	FDA approved	Greater BCVA improvement over sham group in patients with DME over two years	a. Cataract surgery b. Elevation in intraocular pressure c. Glaucoma
IL-6 inhibitor	EBI-031 (Section 3.2.2)	Clinical trial (clinicaltrials.gov ID: NCT02842541) in progress	-	-
IL-6 receptor inhibitor	Tocilizumab (Section 3.2.2)	Clinical trial (READ-4 study, clinicaltrials.gov ID: NCT02511067) in progress	-	-
Integrin inhibitor	Luminate (Section 3.2.2)	Phase 2b trial (PACIFIC, http://www.allegroeye.com/?s=PACIFIC) in progress	Non-inferiority to bevacizumab in mean change in BCVA and CRT for the treatment of DME (phase 2b trial, DEL MAR)	Well-tolerated with no drug toxicity or intraocular inflammation noted (phase 2b trial, DEL MAR)

**Table 3:** Laser photocoagulation for the treatment of DR.

Classification	Drugs	Status for DR Treatment	Clinical Benefits	Adverse Effects
Traditional laser treatments	Focal/grid laser [59]	Adjuvant treatment for DME	Reduce risk of moderate visual loss, increases the chance of visual improvement, decreases the frequency of persistent macular edema (Early Treatment Diabetes Retinopathy Study (ETDRS))	a. Visual acuity loss b. Visual field loss
	PRP [60]	Adjuvant treatment for PDR with high-risk complications	Reduce the rate of severe visual loss in PDR and inhibit the progression of retinopathy (Diabetic Retinopathy Study (DRS))	a. Visual acuity loss b. Constriction of peripheral visual field
New laser approaches	PASCAL [65]	Under clinical evaluation	Precise control of the laser; Decreased treatment time	-
	D-MPL [66]	Under clinical evaluation	Minimize collateral damage	-
	NAVILAS [67]	Under clinical evaluation	High accuracy of laser spots	-

**Table 4:** Other treatments for the treatment of DR.

Classification	Drugs	Status for DR Treatment	Clinical Benefits	Adverse Effects
Cardiolipin inhibitor	MTP-131 (Ocuvia^TM^) [69]	Clinical trial (clinicaltrials.gov ID: NCT02314299) in progress	-	-
Mitochondria specific antioxidant	ALA [70]	Under clinical evaluation	Improved contrast sensitivity in type 1 and type 2 diabetes patients	-
Antioxidant	Lutein [71]	Under clinical evaluation	Visual improvement in DR patients	-

## Abbreviations

ALA	Alpha-lipoic acid
BRB	Blood-retinal barrier
BCVA	Best-corrected visual acuity
CRT	Central retinal thickness
DEX	Dexamethasone
DME	Diabetic macular edema
DR	Diabetic retinopathy
FA	Fluocinolone acetonide
ICAM-1	Intercellular adhesion molecule-1
MCP-1	Monocyte chemotactic protein-1
NAVILAS	Navigated laser system
NPDR	Non-proliferative diabetic retinopathy
PASCAL	Pattern scanning laser
PDR	Proliferative diabetic retinopathy
PRP	Panretinal photocoagulation
SDM	Subthreshold diode micropulse
VCAM-1	Vascular cell adhesion molecule-1
VEGF	Vascular endothelial growth factor
VE-PTP	Vascular endothelial-protein tyrosine phosphatase

## References

[1] Romero-Aroca P., Baget-Bernaldiz M., Pareja-Rios A., Lopez-Galvez M., Navarro-Gil R., Verges R. (2016). Diabetic macular edema pathophysiology: Vasogenic versus inflammatory. J. Diabetes Res..

[2] Gonzalez V.H., Campbell J., Holekamp N.M., Kiss S., Loewenstein A., Augustin A.J., Ma J., Ho A.C., Patel V., Whitcup S.M. (2016). Early and long-term responses to anti-vascular endothelial growth factor therapy in diabetic macular edema: Analysis of protocol I data. Am. J. Ophthalmol..

[3] Brownlee M. (2005). The pathobiology of diabetic complications: A unifying mechanism. Diabetes.

[4] Bek T. (2017). Diameter changes of retinal vessels in diabetic retinopathy. Curr. Diabetes Rep..

[5] Naruse K., Nakamura J., Hamada Y., Nakayama M., Chaya S., Komori T., Kato K., Kasuya Y., Miwa K., Hotta N. (2000). Aldose reductase inhibition prevents glucose-induced apoptosis in cultured bovine retinal microvascular pericytes. Exp. Eye Res..

[6] Romeo G., Liu W.H., Asnaghi V., Kern T.S., Lorenzi M. (2002). Activation of nuclear factor-kappaB induced by diabetes and high glucose regulates a proapoptotic program in retinal pericytes. Diabetes.

[7] Ejaz S., Chekarova I., Ejaz A., Sohail A., Lim C.W. (2008). Importance of pericytes and mechanisms of pericyte loss during diabetes retinopathy. Diabetes Obes. Metab..

[8] Beltramo E., Porta M. (2013). Pericyte loss in diabetic retinopathy: Mechanisms and consequences. Curr. Med. Chem..

[9] Huang H., He J., Johnson D., Wei Y., Liu Y., Wang S., Lutty G.A., Duh E.J., Semba R.D. (2015). Deletion of placental growth factor prevents diabetic retinopathy and is associated with Akt activation and HIF1alpha-VEGF pathway inhibition. Diabetes.

[10] Lupo G., Motta C., Giurdanella G., Anfuso C.D., Alberghina M., Drago F., Salomone S., Bucolo C. (2013). Role of phospholipases A2 in diabetic retinopathy: In vitro and in vivo studies. Biochem. Pharmacol..

[11] Antonetti D.A., Barber A.J., Hollinger L.A., Wolpert E.B., Gardner T.W. (1999). Vascular endothelial growth factor induces rapid phosphorylation of tight junction proteins occludin and zonula occluden 1. A potential mechanism for vascular permeability in diabetic retinopathy and tumors. J. Biol. Chem..

[12] Rousseau S., Houle F., Landry J., Huot J. (1997). p38 MAP kinase activation by vascular endothelial growth factor mediates actin reorganization and cell migration in human endothelial cells. Oncogene.

[13] Li J., Wang J.J., Yu Q., Chen K., Mahadev K., Zhang S.X. (2010). Inhibition of reactive oxygen species by Lovastatin downregulates vascular endothelial growth factor expression and ameliorates blood-retinal barrier breakdown in db/db mice: Role of nadph oxidase 4. Diabetes.

[14] Aiello L.P., Avery R.L., Arrigg P.G., Keyt B.A., Jampel H.D., Shah S.T., Pasquale L.R., Thieme H., Iwamoto M.A., Park J.E. (1994). Vascular endothelial growth factor in ocular fluid of patients with diabetic retinopathy and other retinal disorders. N. Engl. J. Med..

[15] Adamis A.P., Miller J.W., Bernal M.T., D’Amico D.J., Folkman J., Yeo T.K., Yeo K.T. (1994). Increased vascular endothelial growth factor levels in the vitreous of eyes with proliferative diabetic retinopathy. Am. J. Ophthalmol..

[16] Patel J.I., Hykin P.G., Gregor Z.J., Boulton M., Cree I.A. (2005). Angiopoietin concentrations in diabetic retinopathy. Br. J. Ophthalmol..

[17] Rangasamy S., Srinivasan R., Maestas J., McGuire P.G., Das A. (2011). A potential role for angiopoietin 2 in the regulation of the blood-retinal barrier in diabetic retinopathy. Investig. Ophthalmol. Vis. Sci..

[18] Miyamoto K., Khosrof S., Bursell S.E., Rohan R., Murata T., Clermont A.C., Aiello L.P., Ogura Y., Adamis A.P. (1999). Prevention of leukostasis and vascular leakage in streptozotocin-induced diabetic retinopathy via intercellular adhesion molecule-1 inhibition. Proc. Natl. Acad. Sci. USA.

[19] Yuuki T., Kanda T., Kimura Y., Kotajima N., Tamura J., Kobayashi I., Kishi S. (2001). Inflammatory cytokines in vitreous fluid and serum of patients with diabetic vitreoretinopathy. J. Diabetes Its Complicat..

[20] Schroder S., Palinski W., Schmid-Schonbein G.W. (1991). Activated monocytes and granulocytes, capillary nonperfusion, and neovascularization in diabetic retinopathy. Am. J. Pathol..

[21] Miyamoto K., Hiroshiba N., Tsujikawa A., Ogura Y. (1998). In vivo demonstration of increased leukocyte entrapment in retinal microcirculation of diabetic rats. Investig. Ophthalmol. Vis. Sci..

[22] Joussen A.M., Poulaki V., Mitsiades N., Cai W.Y., Suzuma I., Pak J., Ju S.T., Rook S.L., Esser P., Mitsiades C.S. (2003). Suppression of Fas-FasL-induced endothelial cell apoptosis prevents diabetic blood-retinal barrier breakdown in a model of streptozotocin-induced diabetes. FASEB J..

[23] Barouch F.C., Miyamoto K., Allport J.R., Fujita K., Bursell S.E., Aiello L.P., Luscinskas F.W., Adamis A.P. (2000). Integrin-mediated neutrophil adhesion and retinal leukostasis in diabetes. Investig. Ophthalmol. Vis. Sci..

[24] Chibber R., Ben-Mahmud B.M., Coppini D., Christ E., Kohner E.M. (2000). Activity of the glycosylating enzyme, core 2 GlcNAc (beta1,6) transferase, is higher in polymorphonuclear leukocytes from diabetic patients compared with age-matched control subjects: Relevance to capillary occlusion in diabetic retinopathy. Diabetes.

[25] Kasza M., Meleg J., Vardai J., Nagy B., Szalai E., Damjanovich J., Csutak A., Ujhelyi B., Nagy V. (2017). Plasma E-selectin levels can play a role in the development of diabetic retinopathy. Graefe’s Arch. Clin. Exp. Ophthalmol..

[26] Limb G.A., Hickman-Casey J., Hollifield R.D., Chignell A.H. (1999). Vascular adhesion molecules in vitreous from eyes with proliferative diabetic retinopathy. Investig. Ophthalmol. Vis. Sci..

[27] Joussen A.M., Poulaki V., Le M.L., Koizumi K., Esser C., Janicki H., Schraermeyer U., Kociok N., Fauser S., Kirchhof B. (2004). A central role for inflammation in the pathogenesis of diabetic retinopathy. FASEB J..

[28] Suzuki Y., Nakazawa M., Suzuki K., Yamazaki H., Miyagawa Y. (2011). Expression profiles of cytokines and chemokines in vitreous fluid in diabetic retinopathy and central retinal vein occlusion. Jpn. J. Ophthalmol..

[29] Rangasamy S., McGuire P.G., Franco Nitta C., Monickaraj F., Oruganti S.R., Das A. (2014). Chemokine mediated monocyte trafficking into the retina: Role of inflammation in alteration of the blood-retinal barrier in diabetic retinopathy. PLoS ONE.

[30] Koleva-Georgieva D.N., Sivkova N.P., Terzieva D. (2011). Serum inflammatory cytokines IL-1beta, IL-6, TNF-alpha and VEGF have influence on the development of diabetic retinopathy. Folia Med..

[31] Boss J.D., Singh P.K., Pandya H.K., Tosi J., Kim C., Tewari A., Juzych M.S., Abrams G.W., Kumar A. (2017). Assessment of neurotrophins and inflammatory mediators in vitreous of patients with diabetic retinopathy. Investig. Ophthalmol. Vis. Sci..

[32] Abcouwer S.F. (2017). Muller cell-microglia cross talk drives neuroinflammation in diabetic retinopathy. Diabetes.

[33] Sorrentino F.S., Allkabes M., Salsini G., Bonifazzi C., Perri P. (2016). The importance of glial cells in the homeostasis of the retinal microenvironment and their pivotal role in the course of diabetic retinopathy. Life Sci..

[34] Barber A.J., Lieth E., Khin S.A., Antonetti D.A., Buchanan A.G., Gardner T.W. (1998). Neural apoptosis in the retina during experimental and human diabetes. Early onset and effect of insulin. J. Clin. Investig..

[35] Kowluru R.A., Koppolu P. (2002). Diabetes-induced activation of caspase-3 in retina: Effect of antioxidant therapy. Free Radic. Res..

[36] Podesta F., Romeo G., Liu W.H., Krajewski S., Reed J.C., Gerhardinger C., Lorenzi M. (2000). Bax is increased in the retina of diabetic subjects and is associated with pericyte apoptosis in vivo and in vitro. Am. J. Pathol..

[37] Abu-El-Asrar A.M., Dralands L., Missotten L., Al-Jadaan I.A., Geboes K. (2004). Expression of apoptosis markers in the retinas of human subjects with diabetes. Investig. Ophthalmol. Vis. Sci..

[38] Tien T., Zhang J., Muto T., Kim D., Sarthy V.P., Roy S. (2017). High glucose induces mitochondrial dysfunction in retinal muller cells: Implications for diabetic retinopathy. Investig. Ophthalmol. Vis. Sci..

[39] Sasaki M., Ozawa Y., Kurihara T., Kubota S., Yuki K., Noda K., Kobayashi S., Ishida S., Tsubota K. (2010). Neurodegenerative influence of oxidative stress in the retina of a murine model of diabetes. Diabetologia.

[40] Sohn E.H., van Dijk H.W., Jiao C., Kok P.H., Jeong W., Demirkaya N., Garmager A., Wit F., Kucukevcilioglu M., van Velthoven M.E. (2016). Retinal neurodegeneration may precede microvascular changes characteristic of diabetic retinopathy in diabetes mellitus. Proc. Natl. Acad. Sci. USA.

[41] Van Dijk H.W., Kok P.H., Garvin M., Sonka M., Devries J.H., Michels R.P., van Velthoven M.E., Schlingemann R.O., Verbraak F.D., Abramoff M.D. (2009). Selective loss of inner retinal layer thickness in type 1 diabetic patients with minimal diabetic retinopathy. Investig. Ophthalmol. Vis. Sci..

[42] Mitchell P., Bandello F., Schmidt-Erfurth U., Lang G.E., Massin P., Schlingemann R.O., Sutter F., Simader C., Burian G., Gerstner O. (2011). The restore study: Ranibizumab monotherapy or combined with laser versus laser monotherapy for diabetic macular edema. Ophthalmology.

[43] Massin P., Bandello F., Garweg J.G., Hansen L.L., Harding S.P., Larsen M., Mitchell P., Sharp D., Wolf-Schnurrbusch U.E., Gekkieva M. (2010). Safety and efficacy of ranibizumab in diabetic macular edema (resolve study): A 12-month, randomized, controlled, double-masked, multicenter phase ii study. Diabetes Care.

[44] Heier J.S., Korobelnik J.F., Brown D.M., Schmidt-Erfurth U., Do D.V., Midena E., Boyer D.S., Terasaki H., Kaiser P.K., Marcus D.M. (2016). Intravitreal aflibercept for diabetic macular edema: 148-week results from the vista and vivid studies. Ophthalmology.

[45] Gross J.G., Glassman A.R., Jampol L.M., Inusah S., Aiello L.P., Antoszyk A.N., Baker C.W., Berger B.B., Bressler N.M., Writing Committee for the Diabetic Retinopathy Clinical Research Network (2015). Panretinal photocoagulation vs intravitreous ranibizumab for proliferative diabetic retinopathy: A randomized clinical trial. JAMA.

[46] Sivaprasad S., Prevost A.T., Vasconcelos J.C., Riddell A., Murphy C., Kelly J., Bainbridge J., Tudor-Edwards R., Hopkins D., Hykin P. (2017). Clinical efficacy of intravitreal aflibercept versus panretinal photocoagulation for best corrected visual acuity in patients with proliferative diabetic retinopathy at 52 weeks (CLARITY): A multicentre, single-blinded, randomised, controlled, phase 2b, non-inferiority trial. Lancet.

[47] Wells J.A., Glassman A.R., Ayala A.R., Jampol L.M., Aiello L.P., Antoszyk A.N., Arnold-Bush B., Baker C.W., Bressler N.M., Diabetic Retinopathy Clinical Research Network (2015). Aflibercept, bevacizumab, or ranibizumab for diabetic macular edema. N. Engl. J. Med..

[48] Elman M.J., Aiello L.P., Beck R.W., Bressler N.M., Bressler S.B., Edwards A.R., Ferris F.L., Friedman S.M., Glassman A.R., Diabetic Retinopathy Clinical Research Network (2010). Randomized trial evaluating ranibizumab plus prompt or deferred laser or triamcinolone plus prompt laser for diabetic macular edema. Ophthalmology.

[49] Kurihara T., Westenskow P.D., Bravo S., Aguilar E., Friedlander M. (2012). Targeted deletion of vegfa in adult mice induces vision loss. J. Clin. Investig..

[50] Wroblewski J.J., Hu A.Y. (2016). Topical squalamine 0.2% and intravitreal ranibizumab 0.5 mg as combination therapy for macular edema due to branch and central retinal vein occlusion: An open-label, randomized study. Ophthalmic Surg. Lasers Imag. Retina.

[51] Campochiaro P.A., Khanani A., Singer M., Patel S., Boyer D., Dugel P., Kherani S., Withers B., Gambino L., Peters K. (2016). Enhanced benefit in diabetic macular edema from AKB-9778 Tie2 activation combined with vascular endothelial growth factor suppression. Ophthalmology.

[52] Sultan M.B., Zhou D., Loftus J., Dombi T., Ice K.S., Macugen Study G. (2011). A phase 2/3, multicenter, randomized, double-masked, 2-year trial of pegaptanib sodium for the treatment of diabetic macular edema. Ophthalmology.

[53] Lattanzio R., Cicinelli M.V., Bandello F. (2017). Intravitreal steroids in diabetic macular edema. Dev. Ophthalmol..

[54] Boyer D.S., Yoon Y.H., Belfort R., Bandello F., Maturi R.K., Augustin A.J., Li X.Y., Cui H., Hashad Y., Whitcup S.M. (2014). Three-year, randomized, sham-controlled trial of dexamethasone intravitreal implant in patients with diabetic macular edema. Ophthalmology.

[55] Pacella F., Romano M.R., Turchetti P., Tarquini G., Carnovale A., Mollicone A., Mastromatteo A., Pacella E. (2016). An eighteen-month follow-up study on the effects of intravitreal dexamethasone implant in diabetic macular edema refractory to anti-VEGF therapy. Int. J. Ophthalmol..

[56] Campochiaro P.A., Brown D.M., Pearson A., Ciulla T., Boyer D., Holz F.G., Tolentino M., Gupta A., Duarte L., Madreperla S. (2011). Long-term benefit of sustained-delivery fluocinolone acetonide vitreous inserts for diabetic macular edema. Ophthalmology.

[57] Pacella F., Agostinelli E., Carlesimo S.C., Nebbioso M., Secondi R., Forastiere M., Pacella E. (2016). Management of anterior chamber dislocation of a dexamethasone intravitreal implant: A case report. J. Med. Case Rep..

[58] Pearson P.A., Comstock T.L., Ip M., Callanan D., Morse L.S., Ashton P., Levy B., Mann E.S., Eliott D. (2011). Fluocinolone acetonide intravitreal implant for diabetic macular edema: A 3-year multicenter, randomized, controlled clinical trial. Ophthalmology.

[59] Early Treatment Diabetic Retinopathy Study Research Group (1987). Treatment techniques and clinical guidelines for photocoagulation of diabetic macular edema. Early treatment diabetic retinopathy study report number 2. Ophthalmology.

[60] Diabetic Retinopathy Study Research Group (1978). Photocoagulation treatment of proliferative diabetic retinopathy: The second report of diabetic retinopathy study findings. Ophthalmology.

[61] Arnarsson A., Stefansson E. (2000). Laser treatment and the mechanism of edema reduction in branch retinal vein occlusion. Investig. Ophthalmol. Vis. Sci..

[62] Ogata N., Tombran-Tink J., Jo N., Mrazek D., Matsumura M. (2001). Upregulation of pigment epithelium-derived factor after laser photocoagulation. Am. J. Ophthalmol..

[63] Fong D.S., Girach A., Boney A. (2007). Visual side effects of successful scatter laser photocoagulation surgery for proliferative diabetic retinopathy: A literature review. Retina.

[64] Distefano L.N., Garcia-Arumi J., Martinez-Castillo V., Boixadera A. (2017). Combination of anti-VEGF and laser photocoagulation for diabetic macular edema: A review. J. Ophthalmol..

[65] Blumenkranz M.S., Yellachich D., Andersen D.E., Wiltberger M.W., Mordaunt D., Marcellino G.R., Palanker D. (2006). Semiautomated patterned scanning laser for retinal photocoagulation. Retina.

[66] Vujosevic S., Martini F., Convento E., Longhin E., Kotsafti O., Parrozzani R., Midena E. (2013). Subthreshold laser therapy for diabetic macular edema: Metabolic and safety issues. Curr. Med. Chem..

[67] Neubauer A.S., Langer J., Liegl R., Haritoglou C., Wolf A., Kozak I., Seidensticker F., Ulbig M., Freeman W.R., Kampik A. (2013). Navigated macular laser decreases retreatment rate for diabetic macular edema: A comparison with conventional macular laser. Clin. Ophthalmol..

[68] Paradies G., Petrosillo G., Paradies V., Ruggiero F.M. (2009). Role of cardiolipin peroxidation and Ca^2+^ in mitochondrial dysfunction and disease. Cell Calcium.

[69] Alam N.M., Mills W.C.T., Wong A.A., Douglas R.M., Szeto H.H., Prusky G.T. (2015). A mitochondrial therapeutic reverses visual decline in mouse models of diabetes. Dis. Models Mechan..

[70] Gebka A., Serkies-Minuth E., Raczynska D. (2014). Effect of the administration of alpha-lipoic acid on contrast sensitivity in patients with type 1 and type 2 diabetes. Mediat. Inflamm..

[71] Moschos M.M., Dettoraki M., Tsatsos M., Kitsos G., Kalogeropoulos C. (2017). Effect of carotenoids dietary supplementation on macular function in diabetic patients. Eye Vis..

[72] Kan E., Alici O., Kan E.K., Ayar A. (2017). Effects of alpha-lipoic acid on retinal ganglion cells, retinal thicknesses, and VEGF production in an experimental model of diabetes. Int. Ophthalmol..

[73] Li S.Y., Fu Z.J., Ma H., Jang W.C., So K.F., Wong D., Lo A.C. (2009). Effect of lutein on retinal neurons and oxidative stress in a model of acute retinal ischemia/reperfusion. Investig. Ophthalmol. Vis. Sci..

[74] Li S.Y., Fung F.K., Fu Z.J., Wong D., Chan H.H., Lo A.C. (2012). Anti-inflammatory effects of lutein in retinal ischemic/hypoxic injury: In vivo and in vitro studies. Investig. Ophthalmol. Vis. Sci..

[75] Muriach M., Bosch-Morell F., Alexander G., Blomhoff R., Barcia J., Arnal E., Almansa I., Romero F.J., Miranda M. (2006). Lutein effect on retina and hippocampus of diabetic mice. Free Radic. Biol. Med..

[76] McVicar C.M., Hamilton R., Colhoun L.M., Gardiner T.A., Brines M., Cerami A., Stitt A.W. (2011). Intervention with an erythropoietin-derived peptide protects against neuroglial and vascular degeneration during diabetic retinopathy. Diabetes.

[77] Canning P., Kenny B.A., Prise V., Glenn J., Sarker M.H., Hudson N., Brandt M., Lopez F.J., Gale D., Luthert P.J. (2016). Lipoprotein-associated phospholipase A2 (Lp-PLA2) as a therapeutic target to prevent retinal vasopermeability during diabetes. Proc. Natl. Acad. Sci. USA.

[78] Staurenghi G., Ye L., Magee M.H., Danis R.P., Wurzelmann J., Adamson P., McLaughlin M.M., Darapladib D.M.E.S.G. (2015). Darapladib, a lipoprotein-associated phospholipase A2 inhibitor, in diabetic macular edema: A 3-month placebo-controlled study. Ophthalmology.

